# Functional Portability of a Hyperaccumulator-Derived Core Microbiome: Enhancing Cadmium Phytoextraction in *Brassica juncea* L. Through Molecular Reprogramming

**DOI:** 10.3390/toxics14040303

**Published:** 2026-03-31

**Authors:** Lukuan Huang, Shumeng Fu, Shaoting Du, Ying Feng

**Affiliations:** 1 Zhejiang International Joint Laboratory on Low-Carbon Pollution Control and Resource Utilization, Zhejiang Collaborative Innovation Center for Full-Process Monitoring and Green Governance of Emerging Contaminants, Interdisciplinary Research Academy (IRA), Zhejiang Shuren University, Hangzhou 310015, China; 2 College of Natural Resources and Environmental Science, Zhejiang University, Hangzhou 310058, China

**Keywords:** phytoremediation, endophytes, core microbiome, synthetic microbial communities, cadmium

## Abstract

Soil cadmium (Cd) contamination is a persistent threat to global food security, requiring sustainable in situ remediation strategies. While hyperaccumulating plants possess specialized traits for metal extraction, their low biomass limits large-scale application. This study investigates the potential of a core endophytic synthetic community (SynCom-NS)—characterized by heavy metal tolerance and growth-promoting traits, originally derived from the hyperaccumulator *Sedum alfredii*—by assessing its ability to modulate the remediation phenotype of a high-biomass non-host crop, *Brassica juncea*. Pot experiments revealed that SynCom-NS root-zone application significantly alleviated Cd toxicity, increasing total fresh weight by 82% and chlorophyll content by 33%. Crucially, the consortium bypassed the “growth-dilution” trade-off, facilitating a 4.07-fold increase in shoot Cd accumulation. Multi-omics analysis demonstrated a systemic modulation of the host’s defense machinery, marked by a >3-fold surge in glutathione (GSH) levels and the induction of phenylpropanoid biosynthesis for cell wall reinforcement. SynCom-NS application also mediated tissue-specific regulation of the key metal transporter *HMA4*, upregulating its expression in roots to accelerate long-distance translocation while downregulating it in shoots. These findings demonstrate that specialized core microbiomes function as potent bio-inoculants, offering a promising biological strategy for engineering high-efficiency phytoremediation systems.

## 1. Introduction

Global agricultural sustainability is severely threatened by toxic metal contamination, a pervasive environmental crisis exacerbated by rapid industrialization and intensive farming practices. Recent global geospatial analyses reveal that 14% to 17% of arable land is compromised by toxic metal accumulation [1]. Among these ubiquitous pollutants, cadmium represents a paramount concern due to its exceptional soil mobility, high bioavailability, and systemic cellular toxicity. Cadmium accumulation in agricultural soils not only dramatically reduces crop yields but also poses an existential threat to global food security and public health through biomagnification within the human food chain [2]. Consequently, developing efficient and sustainable soil decontamination strategies has become an urgent global imperative.

Phytoremediation, specifically phytoextraction, provides an environmentally friendly and economically viable biological solution for large scale soil remediation [3]. Despite its tremendous theoretical potential, the extensive commercial application of this technology is constrained by a fundamental biological compromise [4]. Hyperaccumulator species such as *Sedum alfredii* possess extraordinary physiological adaptations for extreme metal accumulation and internal detoxification, yet their practical utility in the field is severely limited by remarkably slow growth rates and minimal absolute biomass production [5]. Conversely, rapidly growing high biomass agricultural crops such as *Brassica juncea* inherently lack the requisite genetic mechanisms for heavy metal tolerance, frequently experiencing severe oxidative damage, photosynthetic inhibition, and growth arrest under cadmium stress. Overcoming this inherent physiological limitation between absolute biomass yield and maximum metal concentration remains the central challenge in modern phytoremediation research [6].

Contemporary advances in microbial ecology have catalyzed a paradigm shift toward the plant holobiont concept, which postulates that a host plant and its associated microbiota function collectively as a cohesive evolutionary and ecological unit [7]. Throughout prolonged evolutionary exposure to extreme metalliferous environments, hyperaccumulators selectively recruit specific core endophytic microbes that significantly augment the intrinsic metabolic capabilities of the host [3]. These highly specialized microbial consortia function as an extended phenotype, executing essential ecosystem services that facilitate heavy metal mobilization, oxidative stress alleviation, and systemic cellular detoxification [8].

While early remediation efforts predominantly utilized isolated single strain bioinoculants, these rudimentary applications frequently demonstrated unpredictable efficacy in complex field environments due to intense ecological competition and limited functional redundancy [9,10,11]. Synthetic microbial communities represent a sophisticated bioengineering approach to overcome these limitations, enabling the precise artificial assembly of stable and resilient consortia that effectively mimic the complex architecture of natural core microbiomes [12]. Previous investigations by our research group successfully identified and reconstructed a highly optimized endophytic synthetic community designated as SynCom-NS derived from the natural hyperaccumulator *Sedum alfredii* [13]. This specialized microbial module profoundly enhanced cadmium extraction within its native host. However, it is currently unknown whether these extraordinarily efficient microbial remediation modules are strictly confined by host genotype specificity, or if they can function effectively when applied to phylogenetically distant, naturally susceptible recipient plants [14,15]. The establishment of foreign microbial communities is traditionally impeded by stringent host biochemical selection pressures and intense resource competition with the native soil microbiota [16,17]. However, emerging theoretical frameworks suggest that severe abiotic stresses, such as acute heavy metal toxicity, may drive host physiological responses that permit the functional benefits of specialized exogenous microbes to enhance holobiont survival [18]. Understanding the effects of this microbial application is essential for deploying hyperaccumulator-derived microbiomes to fortify susceptible agricultural crops.

In many agricultural settings, soil Cd concentrations often reach or exceed ~1.0 mg/kg, which is significantly higher than safety thresholds such as the Chinese agricultural soil environmental quality standard (GB 15618-2018) [2,19]. The novelty of the current study lies in empirically testing this cross-species barrier under such realistic stress conditions. We utilized the high-biomass non-host *Brassica juncea* as a recipient chassis to rigorously evaluate the application efficacy of the hyperaccumulator-derived SynCom-NS. By integrating comprehensive physiological phenotyping with high resolution comparative transcriptomics, we seek to elucidate the molecular mechanisms by which an exogenous microbiome restructures the heavy metal homeostasis networks of a susceptible plant. We postulate that successful microbial inoculation will mitigate cadmium-induced phytotoxicity and dramatically amplify systemic metal accumulation, ultimately providing a promising bio-augmentation strategy to resolve the biomass and tolerance limitations in environmental remediation.

## 2. Materials and Methods

### 2.1. Plant Material and Growth Conditions

Seeds of the cadmium accumulating *Brassica juncea* cultivar Xikouhuazi were utilized as the recipient host model. Surface sterilization was performed by immersing the seeds in 75% ethanol for 30 s, followed by three consecutive rinses with sterile distilled water [20]. The sterilized seeds were subsequently sown on 0.5% agar plates and incubated in the dark for 48 h to induce uniform germination.

### 2.2. Synthetic Microbial Community Assembly

The SynCom-NS consortium, comprising four specific core endophytic bacterial strains *Leifsonia shinshuensis*, *Novosphingobium lindaniclasticum*, *Ochrobactrum anthropi*, and *Pseudomonas izuensis*, was prepared for inoculation (Table 1) [13]. Single colonies of each strain were inoculated into individual Luria–Bertani liquid media and cultivated continuously at 30 °C with orbital shaking at 150 revolutions per minute for 72 h. Bacterial cells were harvested via centrifugation, and the supernatant was discarded. The resulting cell pellets were washed three times with sterile water to remove residual media and then resuspended [21]. The optical density at 600 nm of each bacterial suspension was adjusted to 2.0. The final SynCom inoculant was assembled by mixing equal volumes of each standardized strain suspension.

### 2.3. Greenhouse Pot Experiment and Experimental Design

Natural cadmium contaminated soil was collected from agricultural fields in Quzhou City (29.05° N, 119.05° E), with a cadmium concentration of approximately 1.0 mg·kg^−1^. The soil was air-dried, finely sieved, and thoroughly homogenized into a single bulk batch to ensure uniform background cadmium levels before being transferred into experimental pots containing 2.0 kg of soil each [22]. Soil moisture was maintained at approximately 70% of the maximum water holding capacity throughout the experiment [23]. Uniformly developed *Brassica juncea* seedlings were transplanted at a density of three plants per pot. The experimental design consisted of two primary treatments: SynCom-NS inoculation and an uninoculated control, with four independent biological replicates established for each condition. Following transplantation, 5 mL of the prepared SynCom-NS suspension was applied directly to the root-soil interface of each plant every three days. This specific inoculation frequency and volume were selected based on preliminary trials to maintain a stable functional population of the introduced consortium in the rhizosphere against native soil microbiota. The control group received an equal volume of sterile water. Plants were cultivated for 40 days, after which comprehensive destructive sampling of both soil and plant tissues was conducted. Harvested plant tissues were equally divided into three analytical aliquots designated for physiological phenotyping, biochemical assays, and transcriptomic profiling.

### 2.4. Quantification of Heavy Metal Concentrations

To determine metal accumulation, harvested shoot and root tissues were sequentially washed and subsequently immersed in 20 mmol/L Na_2_-EDTA for 15 min to eliminate surface adsorbed heavy metal ions, followed by three rinses with ultrapure water [24]. The tissues were oven dried at 105 °C for 30 min to halt enzymatic activity, then completely desiccated at 65 °C for 72 h until a constant weight was achieved. Approximately 0.1 g of the dried plant material was digested in 6 mL of a concentrated HNO_3_ and H_2_O_2_ mixture at a volumetric ratio of 5 to 1 at 120 °C for 8 h until the solution became transparent. For the determination of total soil cadmium, 0.1 g of air-dried soil passed through a 0.15 mm sieve was digested in 7 mL of a ternary acid mixture consisting of HNO_3_, HClO_4_, and HF at a volumetric ratio of 5 to 1 to 1 at 180 °C for 10 h. Bioavailable soil cadmium was extracted from 5 g of 1.0 mm sieved soil using 10 mL of diethylenetriaminepentaacetic acid extractant agitated at 180 revolutions per minute for 2 h at 25 °C [25]. All digested and extracted samples were diluted with ultrapure water, and cadmium concentrations were quantified using Inductively Coupled Plasma Mass Spectrometry. The bioconcentration factor (BCF) was calculated as the ratio of root cadmium concentration to total soil cadmium concentration, while the translocation factor (TF) was defined as the ratio of shoot cadmium concentration to root cadmium concentration [26].

### 2.5. Biochemical Evaluation of the Plant Antioxidant Defense System

For biochemical analyses, intact plants were carefully excavated from the soil. Fresh leaves were gently wiped, and root systems were thoroughly rinsed with sterile water and blotted dry with sterile filter paper. Approximately 0.2 g of leaf tissue from equivalent developmental nodes and corresponding root segments were collected, placed into homogenization tubes, and immediately snap frozen in liquid nitrogen. Tissues from each plant were divided into three technical replicates for the respective biochemical assays to ensure measurement accuracy. The first sample was utilized for the colorimetric quantification of hydrogen peroxide content. The second sample was dedicated to measuring the levels of reduced glutathione. The third sample was subjected to comprehensive enzymatic profiling to determine the specific activities of core reactive oxygen species scavenging enzymes, including ascorbate peroxidase, catalase, glutathione reductase, peroxidase, and superoxide dismutase [27,28].

### 2.6. RNA Extraction and Comparative Transcriptomics

Fresh leaf and corresponding root tissues were harvested, cleaned, and immediately flash frozen in liquid nitrogen. The frozen samples were pulverized into a fine powder at 65 Hz for 1 min using a pre cooled high throughput tissue homogenizer. Total RNA was extracted utilizing a plant column based total RNA purification kit [29]. Functional annotation of the transcripts was performed against the Gene Ontology, Kyoto Encyclopedia of Genes and Genomes, and NCBI non redundant protein databases. Differential expression analysis was executed using DESeq2 [30]. Transcripts exhibiting an absolute log_2_FC greater than 1 and a statistically significant *p* value of less than 0.05 were classified as differentially expressed genes. Enrichment analyses of the differentially expressed genes for specific biological functions and metabolic pathways were conducted utilizing the topGO version 2.50.0 and clusterProfiler version 4.6.0 software packages.

### 2.7. Quantitative Real Time PCR Validation

To empirically validate the reliability of the RNA sequencing expression profiles, seven candidate genes intrinsically linked to antioxidant defense, photosynthetic capacity, and heavy metal transmembrane transport, including *SOD*, *CAT*, *POD*, *GR*, *GST*, *CHLASE*, and *HMA4*, were selected for quantitative real time PCR analysis (Table 2) [13]. *ACTIN* was employed as the endogenous reference gene for transcript normalization.

### 2.8. Statistical Analysis and Data Visualization

All statistical computations and bioinformatic assessments were performed using R software version 4.1.2. The statistical significance of differences between the uninoculated control and SynCom-inoculated groups was evaluated utilizing an independent Student’s *t*-test, with a significance threshold defined at *p* < 0.05 [31]. Analysis of variance and subsequent post hoc comparisons were executed using the “agricolae” package version 1.3.7. All data visualizations and graphical representations were generated using the “ggplot2” package version 4.0.2.

## 3. Results

### 3.1. Effects of SynCom-NS Inoculation on Host Plant Growth

**Figure 1:**
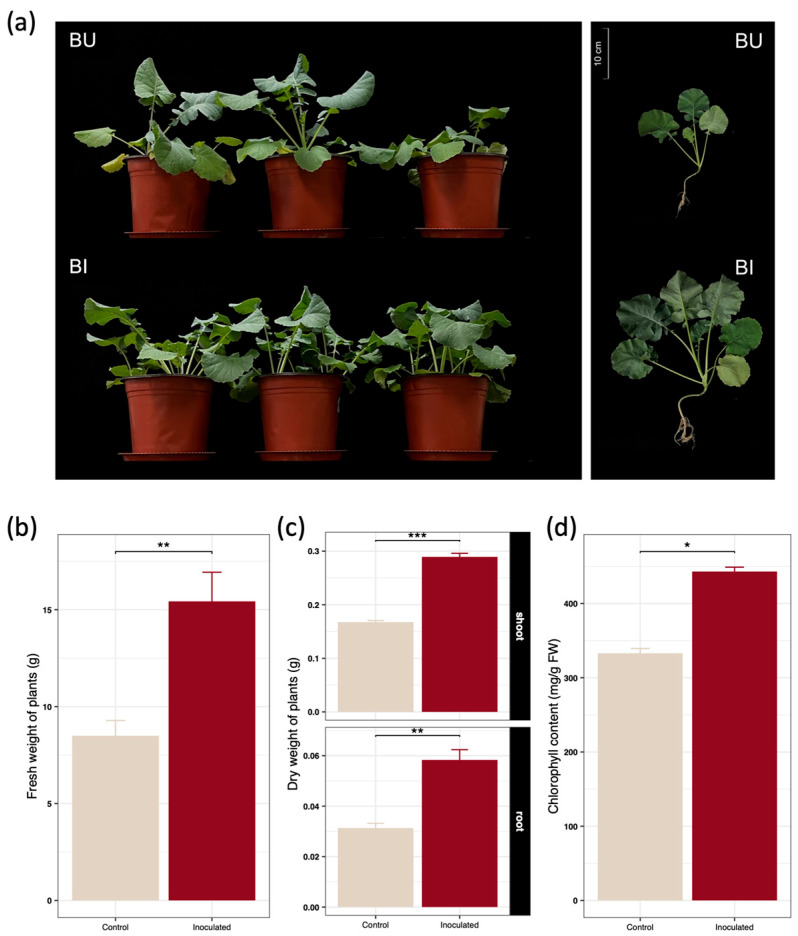
Effects of inoculating SynCom on the (**a**) growth performance (BU: *Brassica* Uninoculated control; BI: *Brassica* Inoculated), (**b**) fresh wight, (**c**) dry weight, and (**d**) chlorophyll content of *Brassica juncea* after 40 days of cultivation. Data are presented as the mean ± standard deviation (*n* = 4). Statistical significance between the inoculated and control groups was determined using an independent Student’s *t*-test. Asterisks indicate significant differences compared to the control: * *p* < 0.05, ** *p* < 0.01, *** *p* < 0.001.

To systematically evaluate the functional efficacy and ecological adaptability of the hyperaccumulator derived synthetic microbial community SynCom-NS within a non-host high biomass crop, a comprehensive series of greenhouse pot trials and high throughput molecular analyses were conducted. *Brassica juncea* seedlings were subjected to natural cadmium contaminated soil conditions under both inoculated and uninoculated treatments. Following the inoculation of the synthetic microbial community SynCom-NS, both the aboveground leaf canopy and the root system of *Brassica juncea* exhibited substantial growth improvements (Figure 1a). The average fresh weight of the inoculated plants reached 15.43 g per plant, representing a 1.82-fold increase compared to the uninoculated control group (Figure 1b). Similarly, the mean dry weights of the roots and shoots under the inoculation treatment were 0.058 g per plant and 0.289 g per plant respectively, which correspond to 1.86-fold and 1.73-fold increases relative to the controls (Figure 1c). These phenotypic enhancements visually and quantitatively demonstrate that the synthetic consortium effectively mitigated the growth retardation symptoms induced by cadmium toxicity. Furthermore, the chlorophyll content in the leaves significantly increased by 33.04% (Figure 1d), indicating that SynCom-NS successfully alleviated cadmium induced chlorosis and protected the photosynthetic apparatus of the host plant.

### 3.2. Influence of SynCom-NS on Cadmium Uptake and Systemic Translocation

**Figure 2:**
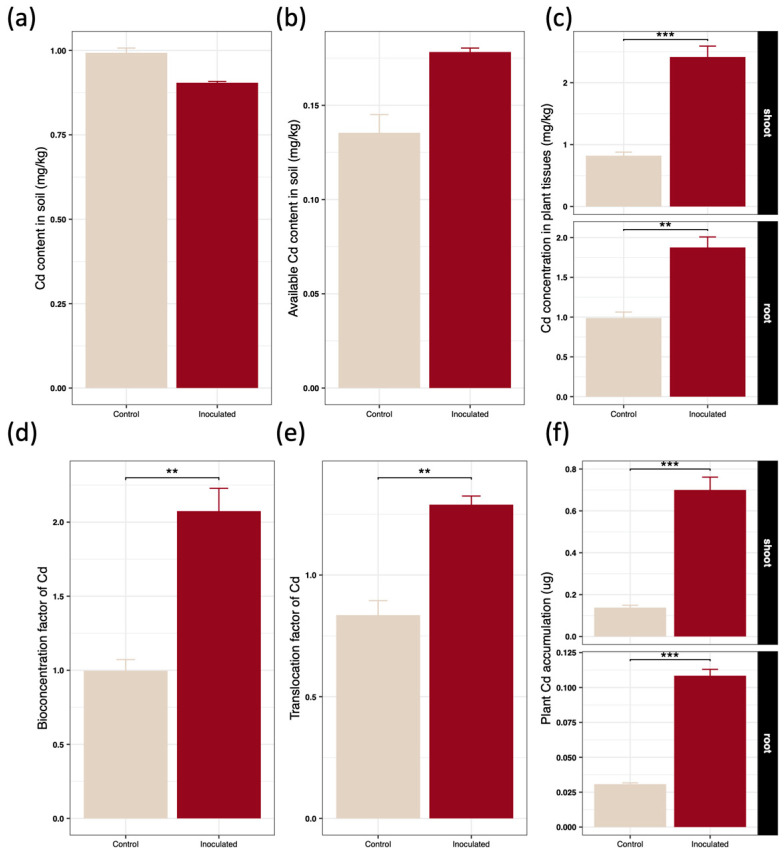
Effects of inoculating SynCom on (**a**) Soil Cd content, (**b**) Soil available Cd content, (**c**) plant Cd concentration, (**d**) Cd bioconcentration factor, (**e**) Cd translocation factor, and (**f**) Cd accumulation in *Brassica juncea* after 40 days of cultivation. Data are presented as the mean ± standard deviation (*n* = 4). Statistical significance between the inoculated and control groups was determined using an independent Student’s *t*-test. Asterisks indicate significant differences compared to the control: ** *p* < 0.01, *** *p* < 0.001.

The application of SynCom-NS led to a reduction in the total soil cadmium content, whereas the bioavailable cadmium fraction in the soil concurrently increased (Figure 2a,b). Concomitantly, the cadmium concentrations in both the roots and shoots of *Brassica juncea* were significantly elevated (Figure 2c). Specifically, SynCom-NS inoculation increased the root cadmium concentration from 0.99 mg·kg^−1^ to 1.88 mg·kg^−1^, and the shoot cadmium concentration surged from 0.82 mg·kg^−1^ to 2.42 mg·kg^−1^. This pronounced elevation confirms that the synthetic community not only facilitated cadmium extraction from the soil but also substantially enhanced its upward translocation within the plant tissues. Calculations of phytoremediation efficiency metrics revealed that the bioconcentration factor of the inoculated plants increased by 108.18%, and the translocation factor improved by 54.34% (Figure 2d,e). Consequently, the absolute cadmium accumulation in the shoots was significantly amplified by 407.37% (Figure 2f), and the root accumulation increased by 252.74% under the bacterial treatment, underscoring the remarkable cross species efficacy of the hyperaccumulator derived microbiome.

To comprehensively evaluate the phytoremediation efficiency and provide a quantitative environmental context, an estimated cadmium mass balance was calculated per pot (Table 3). Although SynCom-NS application drastically amplified the absolute plant Cd extraction, the quantitative mass balance revealed a vast discrepancy between the absolute biological uptake and the apparent soil reservoir reduction. The total mass of Cd effectively removed by the inoculated plant biomass (0.0024 mg) was orders of magnitude lower than the apparent reduction calculated from the final soil concentration (0.19 mg). This discrepancy primarily arises because the final soil Cd concentrations were measured from the rhizosphere—a highly localized microenvironment experiencing intense Cd depletion due to root and microbial activities.

### 3.3. Modulation of the Host Antioxidant Defense System

**Figure 3:**
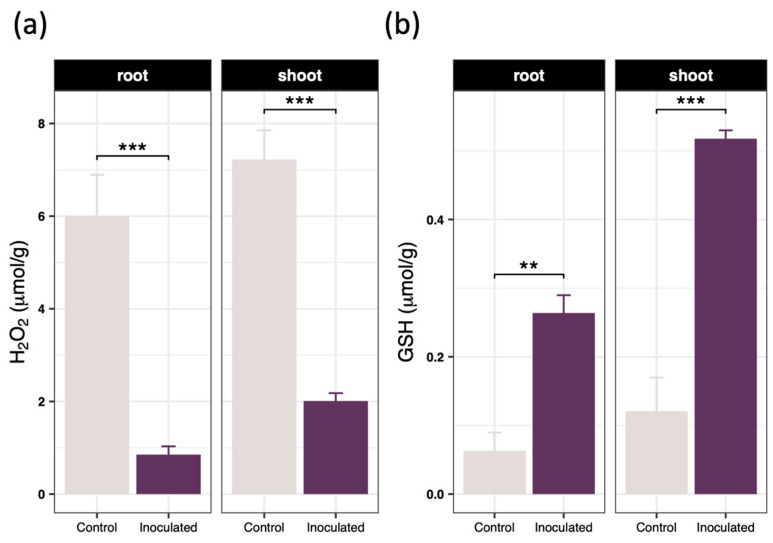
Effects of inoculating SynCom on (**a**) H_2_O_2_ content and (**b**) GSH content in *Brassica juncea* after 40 days of cultivation. Data are presented as the mean ± standard deviation (*n* = 4). Statistical significance between the inoculated and control groups was determined using an independent Student’s *t*-test. Asterisks indicate significant differences compared to the control: ** *p* < 0.01, *** *p* < 0.001.

**Figure 4:**
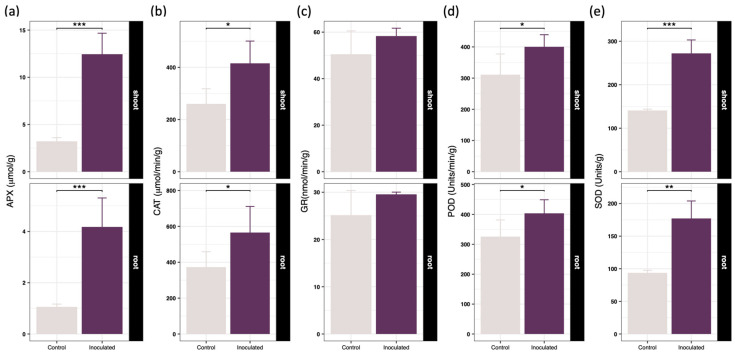
Effects of inoculating SynCom on peroxidase activity in *Brassica juncea* after 40 days of cultivation, including (**a**) APX, (**b**) CAT, (**c**) GR, (**d**) POD and (**e**) SOD. Data are presented as the mean ± standard deviation (*n* = 4). Statistical significance between the inoculated and control groups was determined using an independent Student’s *t*-test. Asterisks indicate significant differences compared to the control: * *p* < 0.05, ** *p* < 0.01, *** *p* < 0.001.

Following bacterial inoculation, the hydrogen peroxide content in the shoots of *Brassica juncea* decreased sharply by 72.18%, accompanied by a substantial 327.71% increase in reduced glutathione levels (Figure 3a,b). A similar biochemical trend was observed in the root tissues, where hydrogen peroxide concentrations dropped by 85.77% and reduced glutathione levels rose by 316.95%. These biochemical shifts clearly indicated that SynCom-NS effectively alleviated oxidative stress and fortified the environmental stress resilience of the recipient host. Comprehensive enzymatic assays revealed a broad enhancement of antioxidant enzyme activities across both tissues (Figure 4). Specifically, the specific activities of ascorbate peroxidase, catalase, glutathione reductase, peroxidase, and superoxide dismutase in the shoots increased by 286.15%, 51.82%, 155.11%, 287.25%, and 93.21% respectively. Correspondingly, these activities in the roots were elevated by 294.82%, 58.67%, 175.32%, 237.52% and 89.07% respectively. These compelling results demonstrate that SynCom-NS significantly upregulated the core reactive oxygen species scavenging enzymes and promoted glutathione biosynthesis, thereby supercharging the detoxification capacity of the host plant.

### 3.4. Comparative Transcriptomics and Quantitative Real Time PCR Validation

**Figure 5:**
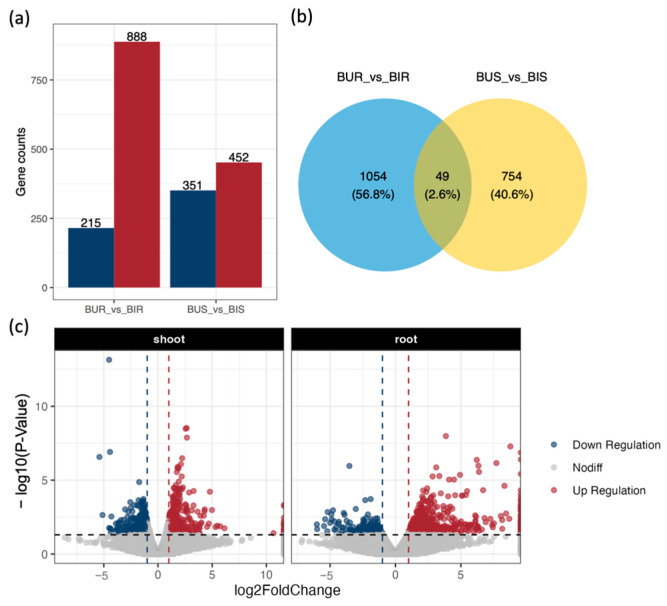
Differential gene responses in *Brassica juncea* after inoculation with SynCom. (**a**) Number of differentially expressed genes among treatment groups; (**b**) Intersection of differentially expressed genes between rapeseed roots and aboveground parts; (**c**) Variation in differential genes between roots and shoots. Genes with an absolute log2FC value greater than 1 and a *p* value less than 0.05 were classified as differentially expressed genes using DESeq2 (*n* = 4).

By mapping these sequences against the non-redundant protein, Gene Ontology, and Kyoto Encyclopedia of Genes and Genomes databases, 99.83% of the total genes were successfully annotated (Table 4). Transcriptomic profiling of the inoculated versus uninoculated plants identified 803 differentially expressed genes in the shoots and 1103 differentially expressed genes in the roots (Figure 5a,c). The root tissues exhibited a dominant upregulation trend with 888 upregulated genes, whereas the shoot tissues were characterized by a higher number of downregulated genes totaling 351. A core set of 49 differentially expressed genes was shared across both tissue types (Figure 5b).

**Figure 6:**
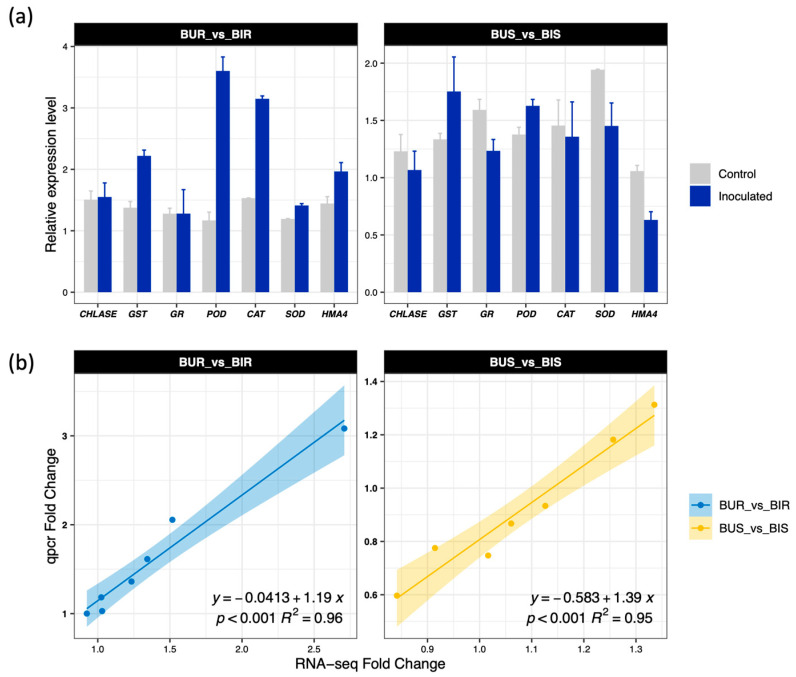
q-PCR verification results of 7 selected genes in *Brassica juncea.* (**a**) Relative expression levels of 7 genes. Data are presented as the mean ± standard deviation (*n* = 4). (**b**) Linear regression of qRT-PCR and RNA-Seq results. The fold change values for the RNA-seq and qRT-PCR of genes are plotted along with the linear fit line. The Pearson’s correlation coefficients (r), coefficients of determination (R^2^), and the *p*-value (two-sided *t*-tests) are shown, respectively (*n* = 4).

To empirically validate the RNA sequencing expression data, quantitative real time PCR analysis was performed on seven selected candidate genes (Figure 6). The validation results confirmed that genes encoding antioxidant enzymes were significantly upregulated in the roots but experienced partial downregulation in the shoots following SynCom-NS inoculation. Furthermore, the heavy metal transporter gene *HMA4* was prominently upregulated in the root tissues and downregulated in the aboveground organs. The expression profiles obtained from the quantitative real time PCR assays exhibited a strong positive correlation exceeding 0.95 with the transcriptomic data, rigorously verifying the high reliability and accuracy of the high throughput sequencing results.

### 3.5. Functional Enrichment Analysis of Differentially Expressed Genes

**Figure 7:**
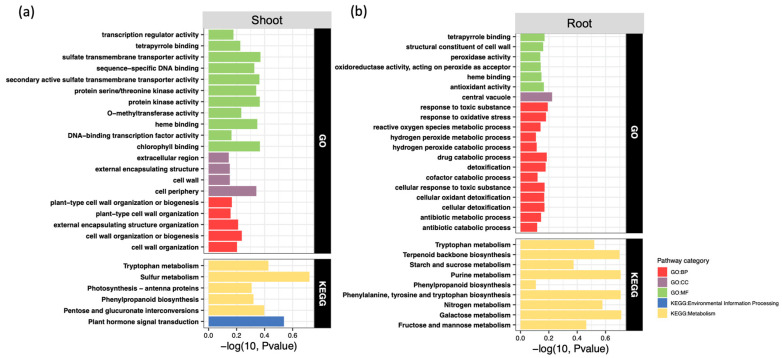
Enrichment analysis of GO and KEGG functional pathways in (**a**) shoot and (**b**) root of *Brassica juncea* after SynCom inoculation. The *x*-axis represents -log_10_ *P*-value, with higher values indicating greater statistical significance of the enrichment. Bar colors denote different pathway categories: red for Biological Process, purple for Cellular Component, green for Molecular Function, blue for KEGG Environmental Information Processing, and yellow for KEGG Metabolism.

Based on the hypergeometric probability distribution *p* values, significantly enriched Gene Ontology and Kyoto Encyclopedia of Genes and Genomes pathways were identified from the tissue specific transcriptomic datasets. The Gene Ontology enrichment analysis of the shoots revealed over 100 significantly enriched pathways, from which the top 20 most significant modules were selected for detailed evaluation. Several critical pathways were structurally related to plant cell wall organization, including the cell wall and plant type cell wall organization modules (Figure 7a). The intense enrichment of these structural pathways facilitates cell wall reinforcement and remodeling, which are paramount for maintaining structural stability, enhancing disease defense, and adapting to extreme environmental pressures. Concurrently, the enrichment of the methyltransferase activity pathway likely accelerated the biosynthesis of crucial secondary metabolites such as lignins and flavonoids. Furthermore, the significant enrichment of chlorophyll binding and heme binding pathways strongly suggests an enhanced photosynthetic efficiency and optimized energy conversion capacity in the shoots following inoculation. The Kyoto Encyclopedia of Genes and Genomes pathway analysis identified six significantly enriched metabolic networks in the shoots, including photosynthesis antenna proteins, phenylpropanoid biosynthesis, pentose and glucuronate interconversions, tryptophan metabolism, plant hormone signal transduction, and sulfur metabolism. The collective enrichment of these networks elucidates the multifaceted role of the synthetic community in boosting photosynthetic yield, antioxidant defense, hormonal homeostasis, and overall stress tolerance in the aboveground tissues.

In the root tissues, the Gene Ontology analysis detected over 200 significantly enriched pathways. A substantial proportion of the top enriched pathways were intricately linked to the plant antioxidant system and oxidative stress response, encompassing the hydrogen peroxide metabolic process, hydrogen peroxide catabolic process, antioxidant activity, and cellular oxidant detoxification (Figure 7b). The prominent activation of these pathways closely correlates with the enhanced reactive oxygen species scavenging efficiency and the subsequent reduction in cellular oxidative damage observed in the physiological assays. Additionally, pathways specifically dedicated to environmental stress responses, such as cellular response to toxic substance, detoxification, response to toxic substance, and central vacuole dynamics, were highly enriched in the roots. The activation of these functional modules directly translates to a heightened adaptability to harmful environmental pollutants. Finally, the Kyoto Encyclopedia of Genes and Genomes analysis of the roots revealed nine significantly enriched major pathways, including phenylpropanoid biosynthesis, starch and sucrose metabolism, fructose and mannose metabolism, tryptophan metabolism, nitrogen metabolism, terpenoid backbone biosynthesis, phenylalanine tyrosine and tryptophan biosynthesis, purine metabolism, and galactose metabolism. These comprehensive transcriptomic signatures suggest that SynCom-NS application strongly modulated the physiological networks governing energy metabolism, root structural growth, and enzymatic antioxidant defense mechanisms within the root system of the non-host plant.

## 4. Discussion

### 4.1. Enhancing Non-Host Metal Tolerance Through Synthetic Community Application

The successful application and subsequent physiological benefits of the hyperaccumulator-derived synthetic community SynCom-NS in the agriculturally important crop *Brassica juncea* demonstrates the immense potential of microbiome-assisted strategies in overcoming the inherent biological limitations of phytoremediation. Conventional phytoremediation strategies are perpetually hindered by a fundamental physiological trade off where plant species possessing extraordinary heavy metal tolerance universally exhibit negligible biomass production, whereas fast growing high biomass crops are intrinsically highly susceptible to metal toxicity [4]. A persistent ecological challenge in microbiome engineering has been the strict host genotype specificity. However, our study demonstrates that severe environmental pressures, such as acute cadmium toxicity, can drive significant holobiont-level adaptations. Under extreme abiotic stress, the urgent requirement for plant survival permits the successful manifestation of the foreign metal remediation module’s benefits, enhancing the host’s overall tolerance without strictly requiring long-term evolutionary coalescence.

The selection of *Brassica juncea* as the non-host recipient chassis was predicated on its exceptional agronomic traits, including rapid vegetative growth, expansive leaf area, and substantial aboveground biomass generation [20,32]. Previous large scale meta-analyses and greenhouse investigations have established that various Brassica species possess an intrinsic baseline capacity for cadmium accumulation, thereby representing premier candidates for engineered phytoextraction provided their heavy metal tolerance can be sufficiently enhanced [33]. A persistent ecological challenge in microbiome engineering has been the strict host genotype specificity that typically restricts the cross-species transfer of microbiome functions [34]. Endophytic communities are highly adapted to the unique biochemical milieu of their native hosts, often resulting in colonization failure when introduced into phylogenetically distant plant species [35]. However, our study demonstrates that severe environmental pressures, such as acute cadmium toxicity, can drive the phenomenon of microbial community coalescence. Under extreme abiotic stress, the urgent requirement for holobiont survival ostensibly overrides the stringent biochemical selection barriers normally imposed by the host genotype, thereby permitting the successful establishment and functional manifestation of the foreign metal remediation module [18].

### 4.2. Microbially Driven Reprogramming of Host Metal Homeostasis and Detoxification

The marked elevation in both root and shoot cadmium concentrations following SynCom-NS inoculation confirms the capability of the synthetic consortium to actively modulate metal bioavailability and transport dynamics within the rhizosphere (Figure 2c). Notably, while the total soil cadmium content decreased over the experimental period, the concentration of bioavailable cadmium in the soil matrix increased significantly (Figure 2a,b). This biogeochemical shift suggests that the introduced microbial community actively secretes specific organic acids, siderophores, and highly affine metal chelating agents that transform immobilized cadmium complexes into readily absorbable bioavailable states [4,25,36]. Furthermore, the microbially induced expansion of the root system architecture, characterized by increased root proliferation and surface area, provided the recipient plant with a vastly larger spatial network for metal interception and absorption [37,38,39]. This enhanced mobilization and uptake mechanism directly mirrors the extended phenotype originally observed in the native hyperaccumulator host *Sedum alfredii*, confirming that highly specialized biogeochemical functions can be seamlessly reconstituted within a naive plant chassis [13,40,41,42].

Elevated intracellular cadmium concentrations typically trigger a devastating cascade of phytotoxic responses, primarily driven by the excessive accumulation of reactive oxygen species that inflict severe oxidative damage upon critical cellular macromolecules and photosynthetic organelles [43]. Uninoculated *Brassica juncea* plants exhibited classic morphological symptoms of heavy metal stress, including pronounced growth retardation, severe foliar chlorosis, and diminished biomass (Figure 1). Conversely, SynCom-NS inoculation dramatically alleviated these phytotoxic symptoms, preserving leaf chlorophyll content and promoting robust systemic growth. As observed in recent biomonitoring studies, the degradation of chlorophyll is a primary physiological indicator of heavy-metal stress and toxicity [44]. Thus, the protection of the photosynthetic apparatus in our inoculated plants underscores the efficacy of the microbial intervention in mitigating systemic cellular damage. Biochemical evaluations revealed a profound microbially mediated fortification of the endogenous antioxidant defense machinery (Figure 3 and Figure 4). The significant suppression of intracellular hydrogen peroxide levels, coupled with the massive systemic upregulation of the non-enzymatic antioxidant reduced glutathione and the enhanced activities of core reactive oxygen species scavenging enzymes, indicates that the synthetic community orchestrates a highly coordinated and efficient detoxification response. This multifaceted defense strategy neutralizes free radicals before they can induce lipid peroxidation or disrupt membrane integrity, thereby significantly enhancing the detoxification capacity and conferring profound metal tolerance to the sensitive host [45,46].

### 4.3. Transcriptomic Signatures of the Extended Phenotype in the Non Host Recipient

High resolution comparative transcriptomics provided fundamental molecular insights into this cross kingdom physiological reprogramming. In the root tissues, the profound enrichment of functional pathways associated with cellular oxidant detoxification, environmental stress responses, and central vacuole compartmentalization provides insights into the potential molecular pathways by which the microbial consortium physically shields the host from metal toxicity (Figure 7b). The prominent activation of secondary metabolite biosynthesis pathways, particularly those governing phenylpropanoids and terpenoids, highlights a sophisticated microbial manipulation of host biochemical networks [47]. These specific secondary metabolites serve crucial protective roles as potent endogenous antioxidants, structural defendants, and metal chelators that mitigate intracellular oxidative stress [48].

In the aboveground tissues, the significant enrichment of cell wall organization and modification pathways implies that the microbial intervention reinforces the physical boundaries of host cells (Figure 7a). This structural remodeling potentially facilitates the sequestration of cadmium ions within the apoplastic space, thereby preventing their entry into the sensitive cytoplasmic environment and safeguarding the photosynthetic apparatus [49,50]. Furthermore, the extensive modulation of crucial plant hormone signal transduction networks, alongside key metabolic pathways such as tryptophan and sulfur metabolism, highlights the capacity of SynCom-NS to actively steer the developmental programming of the host toward a resilient and highly productive physiological state [41,51,52].

### 4.4. Practical Implications, Remediation Targets, and Study Limitations

While this study demonstrates the significant potential of SynCom-NS to augment phytoextraction, several limitations must be acknowledged. First, the initial soil Cd concentration used in this study (~1.0 mg/kg) exceeds the regulatory safety thresholds for agricultural soils (e.g., the Chinese standard GB 15618-2018 limit of 0.3 mg/kg). Although SynCom-NS application achieved a 407.37% increase in absolute shoot Cd accumulation, this pot-based “proof-of-concept” represents a relative efficiency enhancement. For instance, a mass balance estimation reveals that the absolute Cd accumulated in the inoculated plant biomass is minute compared to the apparent reduction in total soil Cd. This discrepancy primarily arises because our final soil measurements were derived from the highly depleted rhizosphere microenvironment, and extrapolating this local depletion to the entire bulk soil matrix overestimates the actual mass removed. Reaching stringent regulatory targets for total mass decontamination will realistically require continuous, multi-season cropping at the field scale.

Furthermore, the current experimental design focused on evaluating the “net holistic effect” of the complete consortium under heavy metal stress. Consequently, it lacked heat-killed bacterial controls, Cd-free soil conditions, and single-strain treatments, limiting our ability to distinctly untangle baseline microbial growth promotion from Cd-specific stress alleviation. Finally, while we observed profound transcriptomic and physiological modulation in the host, this study did not include direct microbial tracking (e.g., 16S rRNA amplicon sequencing) to definitively verify the endophytic establishment and long-term persistence of the introduced strains within *B. juncea*. Future studies will prioritize rigorous microbial tracking, mechanistic controls, and longitudinal field trials to elucidate the exact colonization dynamics and long-term ecological stability of the transplanted microbiomes.

## 5. Conclusions

In summary, this study provides compelling empirical evidence that the functional benefits of hyperaccumulator-derived microbiomes can be leveraged beyond their native host evolutionary history. By utilizing severe heavy metal stress as an ecological context, we successfully applied the core synthetic consortium SynCom-NS to the agriculturally significant and high-biomass crop *Brassica juncea*. This intervention effectively augmented the heavy metal tolerance and phytoextraction capacity of a naive and inherently susceptible recipient chassis. The applied microbial module achieved this by inducing a systemic molecular response in the host, encompassing the fortification of antioxidant defense networks, the structural remodeling of cellular boundaries, and the modulation of transmembrane metal transport pathways. However, it is important to explicitly note that these findings are currently based on pot-scale greenhouse experiments utilizing a single recipient species. Overcoming the physiological trade-off between absolute biomass yield and maximum metal accumulation through targeted microbiome application establishes a promising biological framework for sustainable remediation. Broad applicability to other crops or complex field conditions must be approached with caution, and future research must prioritize multi-species validation and field-scale trials to fully translate this proof-of-concept into large-scale agricultural practice.

## Figures and Tables

**Table 1:** SynCom-NS member strain information and related traits.

Strain ID	Genus and Species	ACC Deaminase	IAA Production (μg/mL)	Cd Resistance (mM)
SaPI1	*Pseudomonas izuensis*	+	55.1	4.0
SaLS1	*Leifsonia shinshuensis*	+	150.0	0.5
SaOA1	*Ochrobactrum anthropi*	−	64.2	0.6
SaNL1	*Novosphingobium lindaniclasticum*	−	53.9	2.0

+: Positive, −: Negative.

**Table 2:** Primers used for qRT-PCR of *Brassica juncea*.

Gene	Forward Primers (5′-3′)	Reverse Primers (5′-3′)
*SOD*	GGTTTCCATGTCCATGCTCT	ATTGTGAAGGTGGCAGTTCC
*CAT*	TCAGCTGCCAGTTAATGCAC	GACAGCAGGTGGAGTTGGAT
*POD*	TTCGAACGGAAAAAGATGCT	AACCCTCCATGAAGGACCTC
*GR*	AAGGCAAAAGAAGGTGCTGA	AGTTCCCTTGCTGGTCTTCA
*GST*	CGTCGTCGAAGAAGAAGAGG	TTTTTGGTGGGAGTTCCAAG
*CHLASE*	GAATATCCGGTGGTGATGCT	TCCGCCGTTGATTTTATCTC
*HMA4*	TCTGTGGCAAAGAAGTAA	ACCAAACTAGACGACCCT
*ACTIN*	CTTGCACCTAGCAGCATGAA	GGACAATGGATGGACCTGAC

**Table 3:** Estimated cadmium mass balance per pot at the end of the 40-day cultivation period.

	Control Group	SynCom-NS Treatment
Initial total soil Cd stock (mg/pot)	2.0	2.0
Final apparent soil Cd stock (mg/pot)	1.99	1.81
Apparent soil Cd reduction (mg/pot)	0.01	0.19
Total plant Cd accumulation (mg/pot)	0.0005	0.0024

**Table 4:** Summary of transcriptome sequencing gene annotation results for *Brassica juncea*.

Annotation Database	Gene Number	Annotation Percentage (%)
NR	96,791	99.83
GO	56,740	58.51
KEGG	32,925	33.95
Total	96,958	99.83

## Data Availability

The raw data supporting the conclusions of this article will be made available by the authors on request.
